# Growth factor receptor-Src-mediated suppression of GRK6 dysregulates CXCR4 signaling and promotes medulloblastoma migration

**DOI:** 10.1186/1476-4598-12-18

**Published:** 2013-03-05

**Authors:** Liangping Yuan, Hongying Zhang, Jingbo Liu, Joshua B Rubin, Yoon-Jae Cho, Hui Kuo Shu, Matthew Schniederjan, Tobey J MacDonald

**Affiliations:** 1 Department of Pediatrics, Aflac Cancer and Blood Disorders Center, Emory University School of Medicine, 2015 Uppergate Drive NE, Atlanta, GA 30322, USA; 2 Department of Pediatrics, Anatomy and Neurobiology, Washington University School of Medicine, St Louis, MO, USA; 3 Departments of Neurology and Neurological Sciences, Stanford University School of Medicine, Stanford, CA, 94305, USA; 4 Department of Radiation Oncology, Emory University School of Medicine, Atlanta, GA, USA; 5 Department of Pathology, Emory University School of Medicine, Atlanta, GA, 30322, USA

**Keywords:** GRK6, CXCR4, Growth factor receptor, PDGFR, Src, Medullobastoma

## Abstract

**Background:**

Metastasis in medulloblastoma (MB) is associated with poor survival. Recent genetic studies revealed MB to comprise distinct molecular subgroups, including the sonic hedgehog (SHH) subgroup that exhibits a relatively high rate of progression. To identify targeted therapeutics against metastasis, a better understanding of the regulation of MB cell migration is needed. G protein-coupled receptor kinases (GRKs) have been implicated in cancer metastasis through their regulation of G-protein coupled receptors (GPCRs) involved in growth factor (GF)-mediated cell migration. However, the specific roles and regulation of GRKs in MB have not been investigated.

**Methods:**

Microarray mRNA analysis was performed for GRKs, GPCRs, and GFs in 29 human MB, and real time RT-PCR was used to detect GRK6 expression in MB cells. Lenti- or retro-virus infection, and siRNA or shRNA transfection, of MB cells was used to overexpress and knockdown target genes, respectively. Western blot was used to confirm altered expression of proteins. The effect of altered target protein on cell migration was determined by Boyden chamber assay and xCELLigence migration assays.

**Results:**

We observed co-overexpression of *PDGFRA*, *CXCR4*, and *CXCL12* in the SHH MB subtype compared to non-SHH MB (5, 7, and 5-fold higher, respectively). *GRK6*, which typically acts as a negative regulator of CXCR4 signaling, is downregulated in MB, relative to other GRKs, while the percentage of *GRK6* expression is lower in MB tumors with metastasis (22%), compared to those without metastasis (43%). In SHH-responsive MB cells, functional blockade of PDGFR abolished CXCR4-mediated signaling. *shPDGFR* transfected MB cells demonstrated increased GRK6 expression, while PDGF or 10% FBS treatment of native MB cells reduced the stability of GRK6 by inducing its proteosomal degradation. Overexpression or downregulation of Src, a key mediator of GF receptor/PDGFR signaling, similarly inhibited or induced GRK6 expression, respectively. siRNA downregulation of *GRK6* enhanced CXCR4 signaling and promoted MB migration, while lentiviral-*GRK6* overexpression suppressed CXCR4 signaling, potentiated the effect of AMD3100, a CXCR4 antagonist, and impaired migration.

**Conclusions:**

Our findings demonstrate a novel mechanism of GF receptor/PDGFR-Src-mediated dysregulation of CXCR4 signaling that promotes MB cell migration, which could potentially be exploited for therapeutic targeting in SHH MB.

## Background

Medulloblastoma (MB) is the most common malignant brain tumor of childhood, and for those with metastasis the prognosis is typically poor [1]. The molecular sub-types of MB have different rates of metastasis, but to date, no clearly defined genetic alteration has been linked to the initiation of metastasis [2-4]. As such, all children with MB receive craniospinal irradiation to treat or protect against the development of metastasis. This treatment is suboptimal and results in excessive neurocognitive morbidity [1]. Thus, identifying the mechanisms of MB migration will provide invaluable insight into MB progression, which ultimately should lead to more effective and less toxic therapeutics for its prevention.

Activation of growth factor (GF) receptor signaling has been implicated in promoting cancer metastasis. Our previous studies described overexpression of the platelet-derived growth factor receptor (PDGFR) in association with metastatic MB, and demonstrated that PDGFR promotes migration through ERK-dependent activation of p21 protein (Cdc42/Rac)-activated kinase 1 (Pak1) signaling [5,6]. More recently, PDGFR overexpression was detected in both the primary and matched metastatic tumors derived from mouse models of MB [7]. Given the prominent association of PDGFR with MB metastasis, we hypothesized that GF receptor signaling, such as that by PDGFR, may also dysregulate other GF-mediated pathways as a mechanism to potentiate cell migration. One such candidate is the chemokine receptor CXCR4, a member of the G protein-coupled receptor (GPCR) family that is closely associated with PDGFR-expressing progenitor cells [8-11]. CXCR4 plays critical roles in the proliferation and migration of granule cell neuron precursors during development, and is involved in cancer metastasis [12-14]. Human stromal cell-derived factor-1α (SDF-1α, also known as CXCL12), binds CXCR4 and activates Gαi-mediated signaling [15]. Upon CXCL12 binding, CXCR4 dimerizes, and is in turn phosphorylated, which induces its internalization and lysosomal degradation resulting in signal termination [15]. Increased CXCR4 expression is associated with aggressive cancer behavior [16]. Cho et al. reported that the MB subgroups molecularly defined as c1 and c3/sonic hedgehog (SHH) are responsible for the majority of relapses and death due to MB progression and that CXCR4 is a marker for the c3/SHH subgroup [17]. A recent study confirmed that SHH signaling is required for CXCR4 activation [18]. The desmoplastic variant of medulloblastoma, characterized by the presence of abundant connective tissue, belongs almost exclusively to the SHH subgroup and makes up about 50% of SHH MB, but predominantly occurs in children less than 3 years of age. Interestingly, SHH MB with desmoplastic histology has a favorable prognosis, whereas non-desmoplastic SHH MB has higher rates of metastasis and an intermediate prognosis [4]. This dichotomy suggests that additional modulators of SHH-CXCR4 activity may determine MB clinical behavior.

G protein-coupled receptor kinases (GRKs) mediate phosphorylation-dependent GPCR internalization and desensitization controlling GPCR activity [19,20]. GRKs selectively phosphorylate activated receptors, promoting high affinity binding of arrestins, which precludes G protein coupling [21,22]. GRKs are divided into three subfamilies: GRK1 (GRK 1 and 7), GRK2 (GRK 2 and 3), and GRK4 (GRK 4, 5 and 6) [23]. Regulation of GPCRs is cell-and GRK-specific. For example, in HEK293 cells, GRK3 and GRK6 promote CXCR4-mediated ERK1/2 activation [15]. However, in HeLa cells, GRK6 induces CXCR4 internalization and inhibits ERK1/2 activation [24]. Similarly, T cells from GRK6-deficient animals are unable to migrate in response to CXCL12 [25], while GRK6 loss in neutrophils enhances CXCL12-induced migration [26]. Evidence indicates that GRK activity is tightly regulated through protein interactions (e.g. Akt or MAPK) that modulate GRK stability [27]. We thus hypothesized that similar mechanisms exist in MB, and report here that GRK-dependent CXCR4 activity is dysregulated by GF receptor/PDGFR-Src signaling resulting in the promotion of MB cell migration.

## Results

### Overexpression of CXCL12 and CXCR4 correlates with SHH MB and PDGFR activity is required for optimal CXCR4 signaling in SHH MB cells

Because of the dichotomy of clinical outcomes observed for SHH MB, we first investigated whether the expression profiles of CXCL12 and CXCR4 in SHH and non-SHH MB is associated with tumor histology (desmoplastic vs. non-desmoplastic) and clinical outcome. We performed microarray RNA analysis on 29 primary human MB specimens and analyzed the gene expression of members of the SHH pathway (to determine SHH from non-SHH MB), *CXCL12* and *CXCR4*. In our cohort, 11/29 (38%) MB analyzed are SHH-active, and the mean age in SHH and non-SHH tumors is 3.7 and 6.4 years, respectively, which are consistent with the frequency and age distributions observed in historical MB populations [1-4]. The percentage of desmoplastic tumors in our cohort (10%) is also consistent with the distribution observed in childhood MB. Consistent with reports by others [2], all of the desmoplastic tumors in our cohort are SHH MB, with desmoplastic tumors representing 27% of the SHH MB (Table 1). All SHH tumors demonstrate co-overexpression of *CXCL12* and *CXCR4*, with the exception of one tumor that was obtained after treatment with high-dose chemotherapy. None of the non-SHH MB display co-overexpression, and only two non-SHH MB demonstrate relative overexpression of *CXCR4* (Table 1). Compared to non-SHH MB, the relative mean expression of *CXCR4* and *CXL12* in SHH MB is 7- and 5-fold higher, respectively (Table 1) (P < 0.01 and P < 0.05), respectively). We also examined the expression of PDGFR pathway members for correlations with SHH MB and found that although *PDGFRA* overexpression is observed in ~50% of the non-SHH MB, the relative mean expression level for *PDGFRA* is 5-fold higher in SHH MB (P < 0.01), and *PDGF-A*, which only binds to PDGFRA, is 4-fold higher (P < 0.01), while that of *PDGF-D*, which only binds PDGFRB, is 3-fold higher (P < 0.01) (Table 2), suggesting that both PDGFRA and PDGFRB are preferentially activated in SHH MB.

**Table 1:** **mRNA Expression Profile of *CXCR4 *and *CXCL12 *in SHH vs. non-SHH MB**

**Tumor histology**	**Collection of specimen**	**Patient age at time of specimen collection**	**Patient status**	**SHH or Non-SHH**	***CXCR4 *relative mRNA level**	***CXCL12 *relative mRNA level**
Desmoplastic	Diagnostic	1 year	DECEASED	SHH	5422	1631
Desmoplastic	Diagnostic	2 years	ALIVE	SHH	1745	217
Desmoplastic	Diagnostic	5 years	ALIVE	SHH	6036	1707
Anaplastic	Post-Rx	3 years	DECEASED	SHH	1216	10
Classic	Diagnostic	9 months	ALIVE	SHH	6225	356
Clssic	Diagnostic	1 year	DECEASED	SHH	2050	567
Classic,	Diagnostic	2 years	DECEASED	SHH	3175	909
Classic	Post-Rx	2 years	DECEASED	SHH	4529	623
Classic	Diagnostic	4 years	ALIVE	SHH	3864	463
Classic	Diagnostic	5 years	ALIVE	SHH	4408	149
Classic, M+	Post-Rx	7 years	DECEASED	SHH	911	2222
				**Mean ± SD**	**3598 ± 1915**	**805 ± 730**
MBEN	Diagnostic	3 years	ALIVE	Non-SHH	138	291
Anaplastic	Diagnostic	2 years	ALIVE	Non-SHH	43	48
Anaplastic	Diagnostic	6 years	ALIVE	Non-SHH	1717	76
Classic	Diagnostic	6 months	ALIVE	Non-SHH	4367	20
Classic	Diagnostic	1 year	ALIVE	Non-SHH	339	200
Classic	Diagnostic	2 years	ALIVE	Non-SHH	107	1144
Classic	Diagnostic	2 years	ALIVE	Non-SHH	240	615
Classic	Diagnostic	2 years	ALIVE	Non-SHH	582	13
Classic	Diagnostic	3 years	ALIVE	Non-SHH	193	92
Classic	Diagnostic	5 years	DECEASED	Non-SHH	185	75
Classic	Diagnostic	6 years	DECEASED	Non-SHH	125	62
Classic	Diagnostic	6 years	ALIVE	Non-SHH	28	43
Classic, M+	Diagnostic	8 years	DECEASED	Non-SHH	198	116
Classic	Diagnostic	11 years	ALIVE	Non-SHH	73	47
Classic	Diagnostic	11 years	ALIVE	Non-SHH	51	59
Classic	Diagnostic	11 years	ALIVE	Non-SHH	374	55
Classic	Diagnostic	12 years	ALIVE	Non-SHH	69	149
Classic	Diagnostic	16 years	ALIVE	Non-SHH	80	73
				**Mean ± SD**	**495 ± 1041**	**177 ± 280**
				**Fold change SHH/Non-SHH**	**+ 7X**	**+ 5X**
**SHH MB desmoplastic vs. Non-desmpplastic**						
Desmoplastic	Diagnostic	1 year	DECEASED	SHH	5422	1631
Desmoplastic	Diagnostic	2 years	ALIVE	SHH	1745	217
Desmoplastic	Diagnostic	5 years	ALIVE	SHH	6036	1707
				**Mean ± SD**	**4401 ± 2321**	**1185 ± 839**
Anaplastic	Post-Rx	3 years	DECEASED	SHH	1216	10
Classic	Diagnostic	9 months	ALIVE	SHH	6225	356
Classic	Diagnostic	1 year	DECEASED	SHH	2050	567
Classic	Diagnostic	2 years	DECEASED	SHH	3175	909
Classic	Diagnostic	2 years	DECEASED	SHH	4529	623
Classic	Diagnostic	4 years	ALIVE	SHH	3864	463
Classic	Diagnostic	5 years	ALIVE	SHH	4408	149
Classic, M+	Post-Rx	7 years	DECEASED	SHH	911	2222
				**Mean ± SD**	**3297 ± 1822**	**662 ± 689**

*SHH MB*, sonic-hedgehog medulloblastoma; *MBEN*, medulloblastoma with extensive nodularity; *M+*, metastatic; *Post-Rx*, post-treatment.

**Table 2:** **mRNA profiles of *GRK*s and *PDGFR/PDGF *in SHH vs. Non-SHH MB**

**SHH MB**							
**Pathological diagnosis**	**Patient status**	***GRK4***	***GRK5***	***GRK6***	***PDGFRA***	***PDGFD***	***PDGFA***
Desmoplastic	DECEASED	122	215	65	941	300	666
Desmoplastic	ALIVE	140	154	56	1071	392	236
Desmoplastic	ALIVE	80	77	60	911	417	192
Anaplastic	DECEASED	49	355	117	48	454	226
Classic	ALIVE	108	78	82	723	65	237
Classic	DECEASED	118	184	86	469	139	391
Classic	DECEASED	134	235	59	2166	231	114
Classic	DECEASED	162	1253	75	926	98	471
Classic	ALIVE	60	265	84	405	80	87
Classic	ALIVE	72	148	93	773	310	91
Classic, M+	DECEASED	101	343	71	767	122	182
	**Mean ± SD**	**104 ± 36**	**301 ± 329**	**77 ± 18**	**836 ± 530**	**237 ± 145**	**263 ± 179**
**Non-SHH MB**							
MBEN	ALIVE	171	245	72	73	155	67
Anaplastic	ALIVE	251	344	69	58	116	102
Anaplastic	ALIVE	272	186	110	21	63	84
Classic	ALIVE	142	624	74	190	10	32
Classic	ALIVE	186	253	76	322	63	111
Classic	ALIVE	192	235	64	406	49	36
Classic	ALIVE	144	666	58	187	52	41
Classic	ALIVE	139	217	71	25	67	125
Classic	ALIVE	143	217	58	43	78	40
Classic	DECEASED	141	293	29	388	100	64
Classic	DECEASED	150	278	51	102	29	16
Classic	ALIVE	252	370	120	145	23	31
Classic, M+	DECEASED	142	454	69	727	79	172
Classic	ALIVE	242	173	70	57	245	35
Classic	ALIVE	207	435	70	49	54	44
Classic	ALIVE	220	731	81	191	58	41
Classic	ALIVE	239	142	42	61	92	84
Classic	ALIVE	285	615	88	7	68	69
	**Mean ± SD**	**195 ± 51**	**360 ± 186**	**71 ± 21**	**170 ± 186**	**78 ± 54**	**66 ± 40**
	**SHH:non-SHH MB**				+**5X**	**+3X**	**+4X**
**SHH desmoplastic vs. desmoplastic MB**							
Desmoplastic	DECEASED	122	215	65	941	300	666
Desmoplastic	ALIVE	140	154	56	1071	65	237
Desmoplastic	ALIVE	80	77	60	911	122	182
	**Mean ± SD**	**114 ± 31**	**149 ± 69**	**60 ± 5**	**974 ± 85**	**162 ± 123**	**362 ± 265**
Anaplastic	DECEASED	49	355	117	48	392	236
Classic	ALIVE	108	78	82	723	417	192
Classic	DECEASED	118	184	86	469	454	226
Classic	DECEASED	134	235	59	2166	139	391
Classic	DECEASED	162	1253	75	926	231	114
Classic	ALIVE	60	265	84	405	98	471
Classic	ALIVE	72	148	93	773	80	87
Classic, M+	DECEASED	101	343	71	767	310	91
	**Mean ± SD**	**101 ± 38**	**358 ± 374**	**83 ± 17**	**785 ± 623**	**265 ± 149**	**226 ± 141**

*SHH MB*, sonic-hedgehog medulloblastoma; *MBEN*, medulloblastoma with extensive nodularity; *M+*, metastatic.

Consistent with the report by Cho et al. [17], we observed that a disproportionate number of patients with SHH tumors died of disease. Only 5/11 (45%) patients with SHH MB are survivors beyond 5 years from diagnosis compared to 15/18 (83%) long-term survivors in the non-SHH group (P < 0.05, two-tailed Fisher’s exact test), (Table 1). In our cohort, there is no significant difference in *MYC* expression between the SHH and non-SHH MB (SHH MB, 645 ± 1678 vs. non-SHH MB, 1032 ± 1756, P > 0.05). With the exception of one case, all the deaths in the SHH MB group were of patients with tumors having non-desmoplastic histology. In the SHH MB, there are no obvious differences in the expression levels of *CXCR4* or *CXCL12* between desmoplastic and non-desmoplastic histology, or between those alive and deceased, although the number of desmoplastic tumors is too small to determine statistical significance. This suggests that if CXCR4 signaling does promote aggressive MB behavior, then post-transcriptional factors regulating CXCR4 activity, rather than *CXCR4* or *CXCL12* expression alone, must dictate this clinical phenotype.

**Figure 1:**
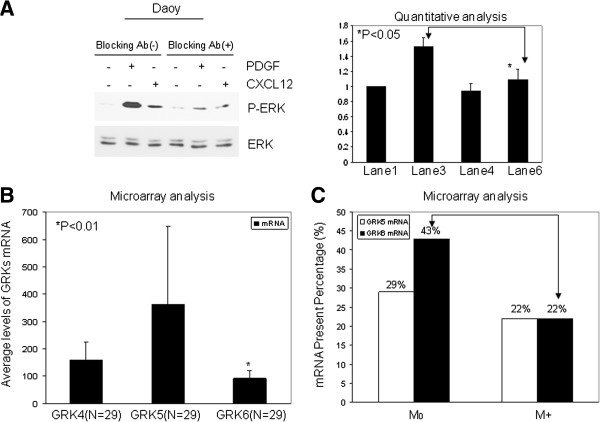
**PDGFR activity is required for optimal CXCR4 signaling in SHH MB cells and GRK6 is downregulated and differentially expressed in MB.** (**A**) Left panel: Daoy cells were starved and treated with or without 1 μg/ml anti-PDGFRβ blocking antibody for 24 h, then stimulated by 10 ng/ml PDGF-BB or 100 ng/ml CXCL12 (SDF-1α) for 15 min. Western blot of P-ERK shows PDGFR function is required for optimal CXCR4 signaling; Right panel: Quantitative analysis of Western blot shows CXCL12-induced P-ERK is significantly decreased by PDGFRβ blocking antibody (P < 0.05, lane 3, 1.53 ± 0.12 vs. lane 6, 1.09 ± 0.15, three independent experiments). (**B**) 29 human MB specimens were collected and used for microarray analysis. Average expression level of *GRK6* mRNA is lowest among the detectable GRKs, (P < 0.01). (**C**) Microarray database of 9 human metastatic and 14 non-metastatic MB showed that the percentage of tumors with detectable *GRK6* mRNA was notably decreased in metastatic MB (M+, 22%), compared to non-metastatic MB (M_0_, 43%). However, the percentage of tumors with detectable *GRK5* mRNA was not appreciably different in M + (22%), vs. M_0_ (29%).

Since we found both *PDGFR* and *CXCR4* overexpression in association with SHH MB, and it has been shown that *PDGF-D* overexpression induces CXCR4 and promotes metastasis in breast cancer cells [14], we postulated that there may be a functional relationship between PDGFR and CXCR4. To test this hypothesis, we used specific anti-PDGFR function blocking antibody to treat Daoy SHH MB cells for 24 h, then stimulated cells with PDGF and CXCL12. Upon CXCL12 ligand binding, CXCR4 activates ERK1/2, thus, CXCL12-induced phosphorylation of ERK (P-ERK) is employed as a downstream marker of CXCR4 activation [24]. Our results show that PDGFR blocking antibody abolished not only PDGF-induced P-ERK, as expected, but also CXCL12-induced P-ERK (Figure 1A) [P < 0.05, lane 3, 1.53 ± 0.12 vs. lane 6, 1.09 ± 0.15 by comparing the densitometry of CXCL12-induced P-ERK/ERK between Daoy cells with or without PDGFR blocking antibody treatment, the ratio of P-ERK/ERK in lane 1 is equal to 1.00 (100%), and the relative changes of ratio of P-ERK/ERK in other lanes were calculated by dividing by the ratio in lane1], indicating that PDGFR activity is required for activation of CXCL12-CXCR4 signaling in SHH-responsive MB cells.

### GRK6 is downregulated and differentially expressed in MB

G protein-coupled receptor kinases (GRKs) initiate GPCR (CXCR4) desensitization by recruiting the binding of β-arrestins to the complex and regulate CXCR4 signaling by mediating the phosphorylation and subsequent internalization of the agonist-occupied receptor [8]. Since we demonstrated that PDGFR activation is required for CXCL12-mediated CXCR4 signaling, we questioned whether PDGFR signaling functions to suppress specific GRK expression and/or activity that would normally act to inhibit CXCR4. To identify the best candidate GRK to test, we first interrogated our microarray dataset of 29 MB and found that *GRK1* and *7* are not expressed by MB; however, among the structurally related GRK4 group consisting of GRK4, 5 and 6, *GRK6* was observed to have the lowest relative expression (P < 0.01, Figure 1B and Table 2). Since our current MB database has too few metastatic MB to make correlations with progression, we re-investigated our previously published MB microarray database of 9 metastatic and 14 non-metastatic MB [5] and found that the percentage of tumors with detectable *GRK6* expression is distinctly lower in those patients with metastatic MB (22%), compared to those with non-metastatic MB (43%), while by comparison, the percentage of tumors with detectable *GRK5* mRNA does not appear different between these groups, suggesting that *GRK6* may be differentially regulated in metastatic MB (Figure 1C). However, because of the small sample size only a trend rather than statistical significance could be observed. *GRK4* was not analyzed in our previous data set. *GRK6* expression is not significantly different between SHH and non-SHH MB, or between histology type, or those alive or deceased in the SHH tumor group. We thus focused on GRK6 in subsequent studies as a candidate GRK that could be involved in GF/PDGFR-mediated regulation of CXCR4 signaling.

### GRK6 expression is negatively regulated by GF receptor/PDGFR at the transcriptional and post-translational level

**Figure 2:**
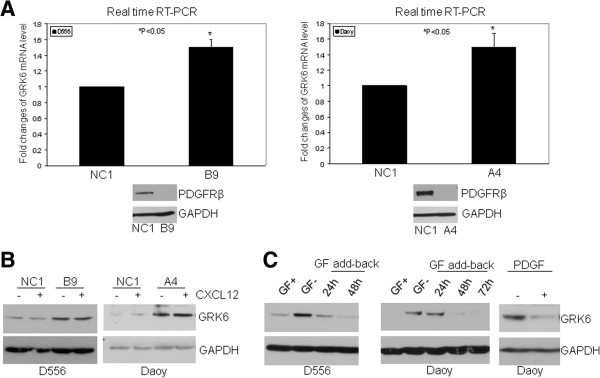
**GRK6 expression is negatively regulated by GF/PDGFR.** (**A**) Quantitative real time RT-PCR was performed to determine the expression of *GRK6* mRNA in medulloblastoma cells with normal vs. downregulated PDGFR expression. (**A**) *GRK6* mRNA is increased 1.5 folds in B9 (PDGFRβ down-regulated by shRNA in D556, shown below left panel) and 1.5 folds in A4 (PDGFRβ down-regulated by shRNA in Daoy, shown below right panel) respectively, compared to the parental cells (D556 NC1 or Daoy NC1), P < 0.05 (N = 3). (**B**) Protein level of GRK6 is increased in D556 (B9) and Daoy (A4) with or without CXCL12 treatment. (**C**) D556 or Daoy was cultured in EMEM medium containing 10% serum (GF+) or serum-free EMEM (GF-) for 24 h, then added 10% serum to the cells cultured in serum-free medium (GF add-back) and cells grown for 24-72 h. Cell lysates were harvested for Western Blot. GF withdrawal results in robust increase of GRK6 in D556 and Daoy (left and middle panels). In right panel, Daoy cells were starved for 24 h and then treated with 10 ng/ml PDGF-BB for 24 h. GRK6 level determined by Western blot shows marked decrease with PDGF treatment.

Since we were unable to detect GRK6 protein by IHC in MB (negative staining, compared to positive control in other tissues, data not shown), we investigated MB cells to determine whether GRK6 expression can be induced, and if so, whether expression is dependent on GF receptor/PDGFR. To address this question, we utilized previously generated MB cells with stable knock-down of PDGFR [6], and then investigated the level of *GRK6* mRNA expression by quantitative real-time RT-PCR. As shown in Figure 2A, *GRK6* mRNA level was significantly higher in the cells with down-regulation of PDGFR (1.5-fold increase in D556 B9 and Daoy A4 PDGFR knock-down cells, compared to the control cells NC1; P < 0.05), indicating that *GRK6* mRNA expression is normally suppressed by PDGFR. To confirm that PDGFR signaling regulates GRK6 protein expression, we used Western blot to evaluate the level of GRK6 in the PDGFR knock-down cells. As shown in Figure 2B, the GRK6 protein level was markedly increased with or without CXCL12 treatment (15 min) in the PDGFR knock-down cells. Although PDGFR is reported to play a role in medulloblastoma, other GF receptors are involved in MB progression. We thus examined whether generalized GF signaling is involved in the regulation of GRK6 expression. Consistently, inhibition of GF receptor activation by GF withdrawal (starvation) similarly increased GRK6 protein levels in MB cells, which could be reversed back to undetectable levels by GF add-back over 48-72 h (Figure 2C, left and middle panels). This indicates that under normal growth conditions, MB GRK6 is suppressed to near undetectable levels. Furthermore, treatment of Daoy cells with PDGF alone for 24 h was sufficient to induce near ablation of GRK6 (Figure 2C, right panel). These data indicate that GRK6 expression is negatively regulated by GF receptor/PDGFR.

**Figure 3:**
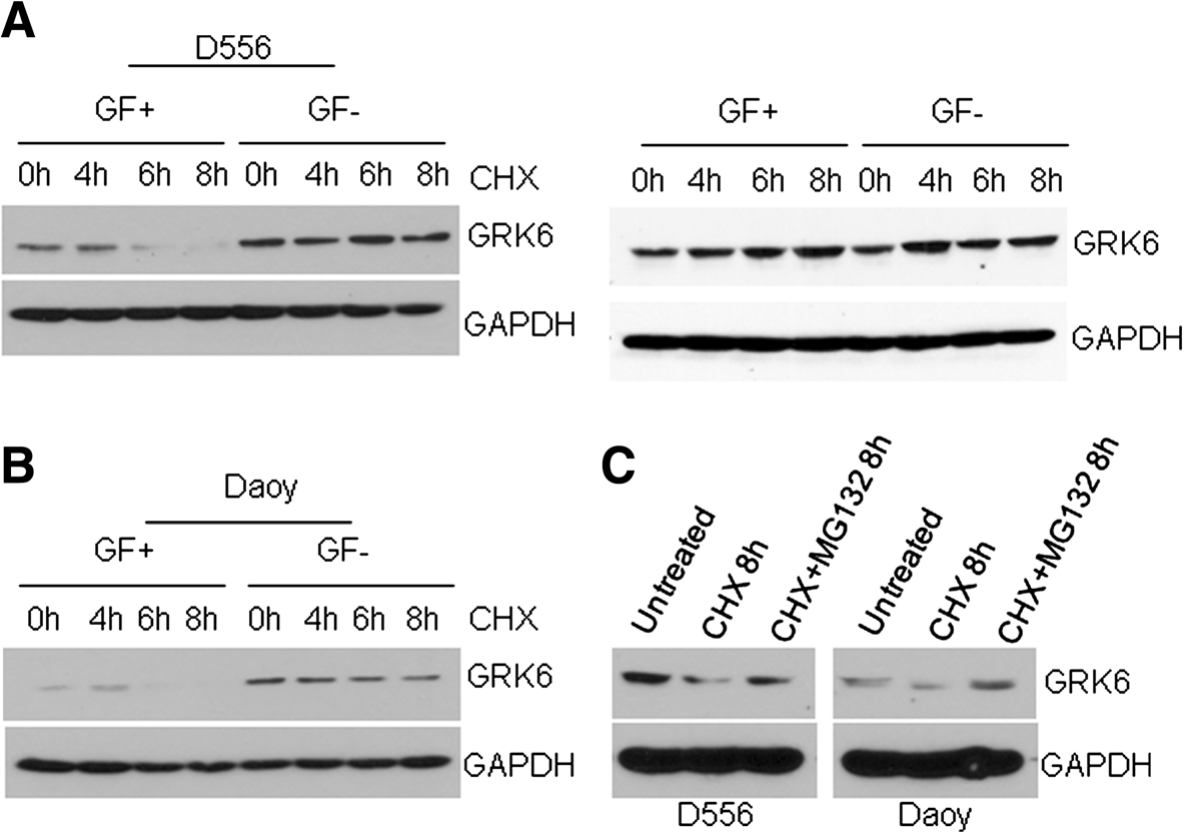
**Growth factor treatment of medulloblastoma cells induces degradation of GRK6 via the proteasomal pathway.** (**A**) D556 or (**B**) Daoy was cultured in EMEM medium with GF + or serum-free EMEM (GF-), treated with100μg/ml CHX or without CHX at indicated time points. GRK6 level was determined by Western blot. Growth factor withdrawal increases stability of GRK6 (**C**) Daoy or D556 was treated with CHX alone or CHX plus 10 μM MG132 for 8 h, then GRK6 protein level was examined by Western blot. GRK6 stability is maintained in the presence of proteasomal inhibitor MG132.

Although PDGFR suppresses *GRK6* mRNA expression, the marked changes in protein expression seemed to indicate that PDGFR induces additional post-translational regulation of GRK6. To examine whether GF activation altered the protein stability of GRK6, we treated MB cells with 100 μg/ml cycloheximide (CHX) at the indicated time points under normal growth conditions or without GF (starved for 24 h prior to CHX treatment). Robust degradation of GRK6 to a near undetectable level was observed in cells growing in normal growth medium at 6 h after CHX treatment; however, GRK6 protein appeared to be relatively stable in the cells grown in the absence of GF at 6-8 h after CHX treatment (Figure 3A, left panel and 3B). No significant difference of GRK6 protein expression was observed between cells growing in normal growth medium and cells in medium without GF at the indicated time points when cells were not treated with CHX to inhibit protein synthesis (Figure 3A, right panel). Similarly, increased GRK6 stability was observed following CHX treatment in the PDGFR knock-down MB cells (not shown). To test whether GRK6 undergoes proteasomal pathway degradation, we treated the cells with and without the proteasome inhibitor, MG132, prior to CHX treatment. As shown in Figure 3C, proteasomal inhibition prevented GRK6 degradation. These data indicate that GRK6 protein stability is negatively regulated under normal growth conditions by GFs, including PDGFR, at the post-translational level through proteasomal degradation.

### GRK6 expression is negatively regulated in a Src-dependent manner

**Figure 4:**
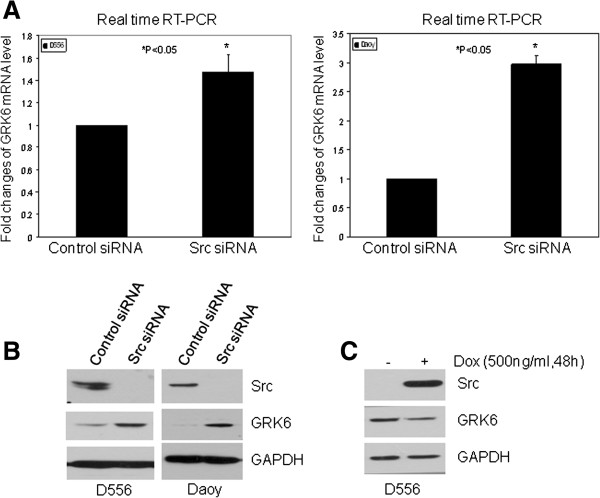
**GRK6 expression is negatively regulated in a Src-dependent manner.** MB cells were transfected with control siRNA or Src siRNA, and then the cells were harvested at 48 h (D556) or 96 h (Daoy) after transfection and examined by real time RT-PCR or Western Blot. (**A**) Real time RT-PCR shows expression of *GRK6* mRNA is significantly increased 1.5 folds in D556 and 3 folds in Daoy by Src siRNA transfection, P < 0.05, respectively. Shown is the representative of three experiments. (**B**) Western blot shows that the protein level of GRK6 is increased by Src siRNA. (**C**) Retro-X Tet on Src-inducible stable cell line was treated by 500 ng/ml Dox for 48 h, and then, Src and GRK6 expression were examined by Western blot. Src- induced expression results in decreased GRK6 protein level.

Src is an important downstream effector of GF receptor/PDGFR signaling and plays a critical role in tumorigenesis [28]. To determine whether Src is involved in the regulation of *GRK6* expression at the transcriptional level, we performed real-time RT-PCR of *GRK6* mRNA in MB cells transfected with control or Src siRNA. As shown in Figure 4A, down-regulation of Src results in a significant increase of *GRK6* mRNA (1.5-fold increase in D556 48 h after transfection, 3-fold increase in Daoy 96 h after transfection). At the protein level by Western blot (Figure 4B), Src downregulation resulted in increased GRK6 levels (48 h in D556 or 96 h in Daoy after transfection). To confirm Src’s role in regulating GRK6 expression, we generated stable MB cells for Doxycycline (Dox)-inducible overexpression of Src. Treatment of stable MB cells with 500 ng/ml Dox for 48 h showed that Src overexpression resulted in decreased GRK6, compared to the same cells without Dox treatment (Figure 4C). Dox treatment had no effect on Src or GRK6 expression in non-transfected parental cells (not shown). Using the Src inhibitor, dasatinib, our data indicate that pharmacologic Src blockade can also induce GRK6 expression (not shown). These data demonstrate that Src, a major downsteam effector of PDGFR and other GF-activated pathways, is sufficient to mediate negative regulation of GRK6 expression.

### CXCR4 signaling and MB cell migration is inhibited by GRK6

**Figure 5:**
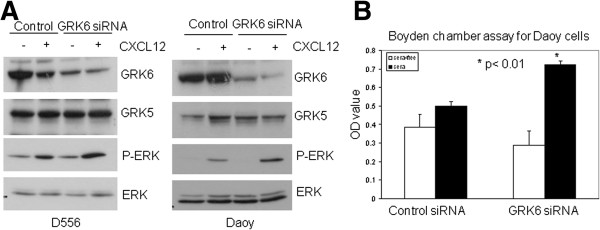
**CXCR4 signaling and MB cell migration is inhibited by GRK6.** (**A**) D556 or Daoy was transfected with control siRNA or GRK6 siRNA. The cells were starved for 24 h after transfection and then stimulated by 100 ng/ml CXCL12 (SDF-1α) for 15 min. The results show that down-regulation of GRK6 causes increased CXCL12-induced P-ERK in both cell lines. (**B**) Daoy was transfected with control siRNA or GRK6 siRNA and then starved for 24 h prior to examination by Boyden chamber migration assay. Results show serum-mediated migration is significantly increased in the cells transfected with GRK6 siRNA (P < 0.01).

To test the function of GRK6 in MB cells, we first used specific GRK6 siRNA to down-regulate GRK6 expression in MB cells and examined the impact of altered GRK6 expression on CXCL12/CXCR4 signaling and cell migration. As shown in Figure 5A, GRK6 down-regulation enhanced CXCL12-mediated phosphorylation of ERK (P-ERK) compared to cells transfected with control siRNA, indicating that GRK6 normally acts to suppress CXCR4 signaling in MB cells. Expression of GRK5, which shares a sequence similar to GRK6, was not affected by GRK6 siRNA transfection. We subsequently demonstrated using Boyden chamber migration assays that serum-mediated cell migration was significantly enhanced in the GRK6 down-regulated MB cells (Figure 5B). Consistent with these results, we found that Src overexpression, which we showed reduces GRK6 expression, similarly promotes cell migration in a scratch assay (data not shown).

**Figure 6:**
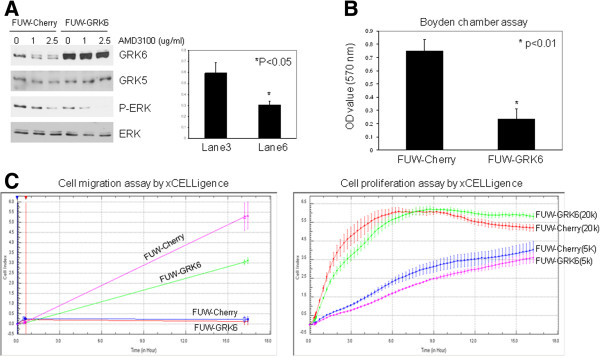
**Overexpression of GRK6 inhibits CXCR4 signaling and cell migration.** (**A**) Left panel: Lentiviral FUW-Cherry (control) or FUW-Cherry-GRK6 (FUW-GRK6)- infected Daoy cells were serum-starved and treated with AMD3100, an antagonist of CXCR4, for 24 h prior to CXCL12 (SDF-1α) stimulation (100 ng/ml for 15 min). The results show that overexpression of GRK6 inhibits CXCL12-induced P-ERK and CXCL12-induced P-ERK is completely abolished by AMD3100 at 2.5 μg/ml in Daoy-FUW-GRK6 cells, not in Daoy-FUW-Cherry cells, that potentiates the CXCR4 inhibitory effect of AMD3100. GRK5 serves as internal control (no change) to demonstrate that effects are GRK6-specific; Right panel: Quantitative analysis of Western blot shows CXCL12-induced P-ERK is significantly inhibited by AMD3100 treatment in Daoy with overexpression of GRK6 [P < 0.05, lane 3, 0.59 ± 0.1 vs. lane 6, 0.31 ± 0.03, the ratio of P-ERK/ERK in lane 1 is equal to 1.00 (100%), three independent experiments]. (**B**) Boyden chamber migration assay shows that serum-mediated migration is significantly decreased in cells with FUW-GRK6 overexpression (P < 0.01), compared to FUW-Cherry control cells. (**C**) xCELLigence was used to monitor real-time cell migration and proliferation. In the left panel, the results show that serum-mediated cell migration is significantly decreased in the cells with FUW-GRK6 overexpression (the upper two lines represent the migration of either FUW-Cherry or FUW-GRK6 in serum-containing medium. The bottom two lines represent the migration of either FUW-Cherry or FUW-GRK6 in serum-free medium). In the right panel, the results show that overexpression of GRK6 has little effect on cell proliferation either at high cell density (2 × 10^4^/well, 20 K) or low cell density (5 × 10^3^/well, 5 K).

To confirm the role of GRK6 in MB cells, we used lentiviral FUW-mCherry (control) and FUW-C-GRK6 to generate stable MB cells overexpressing GRK6. As shown in Figure 6A, CXCL12-mediated P-ERK was decreased in cells with GRK6 overexpression, compared to control cells. Furthermore, treatment of cells with AMD3100, an antagonist of CXCR4, at 2.5 μg/ml completely abolished CXCL12-induced P-ERK in FUW-GRK6, but not in FUW-Cherry cells (Figure 6A, right panel) [P < 0.05, lane 3, 0.59 ± 0.1 vs. lane 6, 0.31 ± 0.03 by comparing the densitometry of CXCL12-induced P-ERK/ERK in Daoy cells with or without overexpression of GRK6. The ratio of P-ERK/ERK in lane 1 is equal to 1.00 (100%)], indicating that GRK6 overexpression potentiates the effect of AMD3100. In Boyden chamber migration assays, we found that MB cell migration was significantly inhibited by overexpression of GRK6 (Figure 6B). Similar results were observed in scratch assays (data not shown). To further validate the role of GRK6 in MB cells, we conducted cell migration and proliferation assays using xCELLigence [29], which allows for monitoring cell activity in real-time. As shown in Figure 6C (left panel), migration in serum-free conditions is similar between FUW-Cherry and FUW-GRK6 cells; however, in the presence of serum, migration is significantly decreased in FUW-GRK6 cells compared to control FUW-Cherry cells. At approximately 16.5 h, the average cell index (number of migratory cells) in FUW-Cherry is 1.7-fold higher than in FUW-GRK6 cells (P < 0.01). To assess whether the difference in migration was the result of differences in cell viability and proliferation, we simultaneously performed a proliferation assay at the beginning of the migration assay. The results showed no significant difference in the proliferative capacity of the control and GRK6 overexpressing cells at either high (2 × 10^4^/well) or low (5 × 10^3^/well) cell density (Figure 6C, right panel). Together, these data indicate that in these MB cells GRK6 functions to suppress CXCR4 signaling and inhibit cell migration, and thus optimal signaling and migration is maintained through ongoing suppression of GRK6 levels and activity.

## Discussion

Children diagnosed with sonic hedgehog-activated MB (SHH MB) displaying desmoplastic histology have a good prognosis, while those with non-desmoplastic histology have higher rates of metastasis and an intermediate prognosis, indicating that additional factors to SHH activation account for the clinical dichotomy observed [2-4,17]. Recent evidence shows that CXCR4 signaling, which is critical to the proliferation and migration of granule neuron precursors during development, is dependent on SHH for its activation in MB [13,18,30,31]. This finding, coupled to CXCR4’s reported role in tumor progression [14,16,32,33], point towards dysregulated CXCR4 signaling as a possible key determinant of SHH MB clinical behavior. We observe high co-expression levels of *CXCR4* and *CXCL12*, as well as *PDGFRA*, distinctly associated with SHH MB. Herein, we demonstrate that GF receptor/PDGFR and Src, a major GF/PDGFR downstream effector, act to suppress the expression and stability of the G protein-coupled receptor kinase GRK6 in MB cells, which in turn functions to maintain CXCR4 signaling and promote cell migration, thereby identifying a new mechanism for the dysregulation of this signaling axis in MB. This finding has important implications for our understanding of SHH MB clinical behavior and potential translation to therapeutic targeting.

GPCRs are desensitized by agonist-induced GRK-mediated phosphorylation, whereby the receptors are uncoupled from heterotrimeric G-protein signaling. Suppression of GRKs, and loss of GRK-mediated desensitization, can result in the prolonged activation of GPCRs. The involvement of CXCR4 in cancer metastasis appears to be due to dysregulation of the receptor leading to enhanced CXCR4 signaling [14,24]. In breast cancer cells, a similar functional relationship between the PDGFR pathway and CXCR4 has been reported, whereby overexpression of PDGF-D, which specifically binds to and activates PDGFRB, was shown to induce CXCR4 expression and promote lymph node metastasis [34]. GRKs can also regulate EGFR and PDGFR activity, and in turn, GRKs may be regulated at the mRNA and protein level by altered oncogenic receptor signaling [35].

The description of GRK6 expression and its functional role in cancer is very limited, and until now, has not been reported in MB. In this study, we found that the percentage of *GRK6* expression is lower in MB tumors with metastasis (22%), compared to those without metastasis (43%); however, these data revealed only a trend in MB, with the difference not being statistically significant due to the small sample size. GRK6 typically has a negative regulatory role in CXCR4 activation and CXCL12-induced cell migration [24,26]. For example, GRK6 deficiency is associated with impaired desensitization and enhanced CXCR4-mediated neutrophil migration and has been implicated in the pro-inflammatory response seen in rheumatoid arthritis [26,36]. However, in HeLa cells, siRNA-based functional screening identified GRK6 as a critical positive regulator of integrin-mediated cell adhesion and migration [37]. Similarly, GRK6 silencing in myeloma cells induced a tumor suppressor effect by inhibiting STAT3 phosphorylation and decreasing tumor cell survival [38].

To date, we have little knowledge regarding the regulation of GRK6. Herein, we demonstrate that GF/PDGFR-Src activation results in decreased expression of GRK6 at the transcriptional and post-translational level to maintain optimal CXCR4 signaling. In fact, we found that Src, a key mediator of PDGFR signaling and other GF-induced pathways [28], can independently regulate GRK6 expression, indicating that Src could be a critical therapeutic target in MB, especially given its additional role as a central node in other pro-migratory and pro-survival signals. This therapeutic potential is further illustrated in our study, which shows that targeting CXCR4 with the inhibitor AMD3100, can be potentiated by the overexpression of GRK6. Since we show that Src suppresses GRK6, Src inhibitors could potentially be used to elevate levels of suppressive GRK6. Given that we have previously shown that PDGFR can regulate Rac1-Pak1 signaling important for cytoskeletal rearrangements required for MB cell migration [6], it remains to be seen whether the combined inhibition of PDGFR-Src-CXCR4 may act synergistically to suppress SHH MB growth and progression. In our study, the observation that specific alteration of GRK6 did not itself impact MB cell growth indicates that GRK6 is a critical mediator of GF receptor/PDGFR-Src oncogenic signaling for CXCR4-mediated migration, but is not essential for maintaining CXCR4-mediated growth. Rather, other mechanisms, and perhaps other GRKs may be necessary to regulate growth. Although we focused on GRK6 in this study because of its apparent dysregulated expression in metastatic MB, it is possible that other GRKs that we found expressed by MB (i.e. GRK4 and 5) may also play a role in MB growth and progression. For example, PDGFR/Src has been shown to regulate GRK2 activity in other cell types and the suppression of GRK3 appears necessary for maximal glioblastoma cell growth [35,39]. Further studies will be necessary to investigate the potential functional role and regulation of GRK4 and GRK5 in MB as well as the effect of targeting GRKs and PDGFR-Src dysregulation of the CXCR4 signaling axis on MB progression *in vivo*.

## Conclusion

In summary, we found that GF receptor/PDGFR-Src-mediated suppression of GRK6 acts to promote CXCR4 signaling and cell migration irrespective of CXCL12 ligand and demonstrate a novel mechanism of GF receptor/PDGFR-Src-mediated dysregulation of CXCR4 signaling that promotes MB cell migration, and thus targeting this axis in SHH MB could represent a potent new therapeutic strategy for MB.

## Methods

### Cell culture and reagents

Daoy and D556 human medulloblastoma cells were cultured in EMEM with 10% fetal bovine serum (FBS). CXCL12 and anti-human PDGFRβ antibody (blocking antibody, cat# AF385) were purchased from R & D Systems (Minneapolis, MN).

### Patient samples

MB frozen tumor specimens were consented for and obtained from the Children’s Healthcare of Atlanta (CHOA) (n = 29) tumor tissue repository. The research protocols and amendments were approved by the institutional review boards of CHOA, Emory University, and the CHOA tumor bank committee. All tumor specimens were studied as deidentified tissue samples and reviewed as part of this study by board certified neuropathologist (MS) according to WHO criteria (2007).

### Expression profiling

RNA was extracted from 29 frozen MB tumor samples using the Trizol reagent (Invitrogen, Carlsbad, CA) and was profiled by AROS Biosciences on the Affymetrix human genome U133 Plus 2.0 array with the 3^′^ IVT Express Labeling Kit (Affymetrix, Santa Clara, CA). CEL files were preprocessed using RMA [40] and probesets collapsed to genes using the Genepattern software suite (http://www.broadinstitute.org/cancer/software/genepattern/). Sample were then assigned to a molecular subgroups as previously described, using a classifier based on support-vector machines [17]. In addition, the database from previously published microarray expression profiling of medulloblastoma [5] was used to supplement mRNA profiling data analysis of metastatic vs. non-metastatic medulloblastoma. The relative mean expression level listed for each gene is calculated by the Affymetrix software.

### Western blot

Western blot of whole cell lysates harvested in lysis buffer (Cell Signaling Technology, Danvers, MA) was performed with the following primary antibodies: GRK5, GRK6 and PDGFRβ (Santa Cruz, CA); phospho-PDGFRβ (Tyr751), phospho-ERK and ERK1/2 (Cell Signaling Technology). Goat anti-mouse or rabbit horseradish peroxidase secondary antibodies (Santa Cruz) were used and the immunoreactive bands were detected by ECL. Densitometric analysis of the visualized bands was used to quantitate and compare the relative changes in levels of target proteins.

### Real time RT-PCR

Total RNA was prepared from human MB cell lines (Daoy or D556). Random-primed single-stranded cDNA was made from total RNA by using the Superscript III kit (Cat# 18080-051, Invitrogen, Carlsbad, CA). The following oligonucleotides as primers were used for real time PCR: Glyceraldehyde-3-phosphate dehydrogenase (GAPDH), 5′- CGTGCCGCCTGGAGAAACC-3′ (forward), 5′TGGAAGAGTGGGAGTTGCTGTTG-3′ (reverse). Human GRK6 primers were purchased from Qiagen (cat# QT00043295). GRK6 cDNA was amplified by real-time PCR (35 cycles: 95°C for 10 min, 94°C for 1 min, 55°C for 1 min, 72°C for 1 min, extension 72°C for 8 min) in real-time cycler from Applied Biosystems (Foster City, CA). Each sample was set at least in triplicate for each PCR. Data analysis was performed according to the absolute standard curve method. Data are presented in relation to the respective housekeeping gene and normalized the fold change of control cells to 1, then calculated the relative fold changes.

### SiRNA and shRNA transfection

Human GRK6 ON-TARGETplus SMARTpool siRNA (L-004627-00-0005), Src ON-TARGETplus SMARTpool siRNA (L-003175-00-0005) and negative control non-targeting siRNA (D-001810-01-05) were purchased from Dharmacon (Chicago, IL). Each SMARTpool is a mixture of 4 siRNA sequences with advantages in both efficacy and specificity. For siRNA transfections, 1.5 × 10^5^ cells were seeded in each well of a six-well plate and grown to 50-60% confluency prior to transfection. Cells were transfected with siRNA using Lipofectamine 2000 (Invitrogen, Carisbad, CA) for 48-96 h according to the manufacturer’s instruction and then cells were treated and harvested for further experiments. The stable shPDGFR and control shRNA cells (Daoy and D556) used in these experiments were previously generated by us as described [6].

### GRK overexpressing cells

The lentiviral FUW-mCherry and FUW-C-GRK6 were kindly provided by Dr. Joshua Rubin (Washington University in St Louis, MO). To make stable medulloblastoma cell lines with overexpression of GRK6, 4 × 10^5^ cells were seeded in 60 mm dishes before infection and then, lentiviral FUW-mCherry and FUW-C-GRK6 were added into each dish for 6 h. Media was changed and cells grown for 2 or 3 days before cell-sorting per the flow cytometry core facility protocols to separate sort-positive and sort-negative cells. Western Blots were performed at least three times to verify the stable cells.

### Src overexpressing inducible cells

The plasmid pRetro HA-Src A was kindly provided by Dr. Hui Kuo Shu (Emory University, Atlanta, GA). Briefly, pCSrc A HA14 plasmid was first digested with Xho1, filled in with T4 polymerase and finally digested with BamH1. The excised HA-Src A cDNA was inserted into the cloning sites between BamH1 and Nru1 of pRetroX-Tight-Pur (Clontech, Mountain View, CA, USA). The regulatory vector and the pRetro HA-Src A were transfected into phoenix-ampho package cells and then the supernatant was harvested to infect MB cell lines. The cells were selected by G418 plus puromycin after infection. 500 ng/ml doxycycline (Dox) was added to induce expression of Src. The overexpression of Src was identified by Western Blot.

### Boyden chamber migration assay

Fibronectin-mediated cell adhesion and migration was assessed using the QCM-FN Quantitative Cell Migration Assay (cat# ECM580, Millipore). Briefly, cells were cultured to 80% confluency and harvested with trypsin/EDTA and resuspended in serum-free EMEM. 1.0 × 10^5^ cells in 200 μL serum-free EMEM were seeded into the pre-coated (fibronectin or BSA) upper chambers. The lower well contained 500 μL EMEM with 10% FBS. Each chamber was set in triplicate. The cells were incubated for 4-5 h at 37°C, stained with cell staining solution and then washed in distilled water. Cells were eluted with extraction solution and 100 μL transfered to a 96-well microtiter plate for absorbance reading (570 nm).

### xCELLigence migration and proliferation assays

xCELLigence instrumentation and protocols for the measurement of real-time migration and proliferation were provided by Roche Diagnostics Corporation. xCELLigence is an electrical impedance-based system that allows for the measurement of real-time cell migration and proliferation [29]. We used CIM plate (Cat# 05665817001, Roche) for migration assay and the E-plate (Cat# 05469830001, Roche) for proliferation assay. Briefly, for migration assay, we seeded 4 × 10^4^ cells into each chamber and set 4 chambers for each cell type. After all chambers were set up, the CIM plate was put into xCELLigence instrument at 37°C, 5% CO2 incubator for migration assay. Similarly, we seeded 2 × 10^4^ cells or 5 × 10^3^ cells into each well and set up 4 wells for each cell type, then ran proliferation assay in xCELLigence instrument at 37°C, 5% CO2 incubator. The impedance was recorded in 15 min intervals.

### Statistical analysis

Group differences and p-values were calculated using Student’s *t*-test. Difference of group percentage was calculated using Fisher’s exact test. P values < 0.05 were considered as statistically significant.

## Abbreviations

PDGFR: Platelet-derived growth factor receptor; SHH: Sonic hedgehog; GPCR: G protein-coupled receptor; GRK: G protein- coupled receptor kinase; SDF-1α: Human stromal cell-derived factor-1α; CXCR4: C-X-C chemokine receptor type 4.

## Competing interests

The authors declare that they have no competing interests.

## Authors’ contributions

LY participated in the design of the study, generated stable cell lines overexpressing GRKs, conducted Western blots, siRNA transfections, proliferation and migration assays, performed statistical and data analysis, and drafted the manuscript. HZ generated stable cell lines overexpressing Src, conducted real time RT-PCR and corresponding Western blots. JL performed PCR primer design, real time RT-PCR and corresponding statistical analysis. JBR aided in study design, data analysis and interpretation, critical revision of the manuscript, and provided the GRK lentivirus. YJC performed the microarray gene expression analysis. HKS generated pRetro HA Src A plasmid in his laboratory and provided the plasmid and technical assistance for generation of stable cell lines. MS provided neuropathology interpretation for all tissue specimens. TJM conceived the research, directed all experiments and provided the oversight for all data analysis, results interpretation and the draft of the final manuscript. All the authors have read and approved the final manuscript.
